# Antimicrobial Resistance of *Staphylococcus borealis* Isolated from Pig Farms: High Prevalence of SCC*mec* Type V and Emergence of *cfr*-Positive Isolates

**DOI:** 10.3390/antibiotics14090910

**Published:** 2025-09-09

**Authors:** Ji Hyun Lim, Ji Heon Park, Gi Yong Lee, Soo-Jin Yang

**Affiliations:** 1 Department of Veterinary Microbiology, College of Veterinary Medicine and Research Institute for Veterinary Science, Seoul National University, Seoul 08826, Republic of Korea; 2 Department of Molecular Microbiology and Immunology, Keck School of Medicine, University of Southern California, Los Angeles, CA 90033, USA

**Keywords:** *Staphylococcus borealis*, pig farms, antimicrobial resistance (AMR), SCC*mec* V, *cfr*

## Abstract

Background: The emergence of livestock-associated antimicrobial-resistant staphylococci, particularly non-*aureus* staphylococci, has become a major public health problem requiring immediate global attention. Methods: In this study, 92 *Staphylococcus borealis* isolates from 20 different pig farms in Korea were examined to determine the following: (1) antimicrobial-resistance (AMR) profiles of the isolates, (2) prevalence of methicillin resistance and staphylococcal cassette chromosome methicillin resistance gene (SCC*mec*) types, (3) occurrence of chloramphenicol–florfenicol resistance gene (*cfr*)-mediated oxazolidinone resistance, and (4) genomic characteristics of *cfr*-positive methicillin-resistant *S. borealis* (MRSB) via whole-genome sequence (WGS) analysis. Results: The overall rate of *S. borealis* isolation was 9.1% (92 isolates/1009 swabs), and 34.8% (32/92) of the isolates were MRSB. Surprisingly, all 32 MRSB isolates carried SCC*mec* V for methicillin resistance, and 31/32 MRSB isolates displayed multidrug-resistance phenotypes. Although 22 *cfr*-positive *S. borealis* isolates (20 MRSB and two methicillin-susceptible *S. borealis*) were identified, most of the isolates were susceptible to linezolid because they carried the 35-bp insertion sequence in the *cfr* promoter. Moreover, WGS analyses suggested horizontal transmission of SCC*mec* V and *cfr*-containing plasmids among different staphylococci species, including *Staphylococcus aureus*, *S. epidermidis*, and *S. borealis*. Conclusions: To the best of our knowledge, this study is the first to describe the AMR characteristics of livestock-associated *S. borealis* isolates, particularly the high prevalence of SCC*mec* V and *cfr*. Collectively, these results suggest that *S. borealis* is a crucial reservoir of AMR genes on pig farms in Korea.

## 1. Introduction

Coagulase-negative staphylococci are common commensal inhabitants of the skin and mucous membranes in humans and various animals. Although outbreaks of livestock-associated (LA) methicillin-resistant *Staphylococcus aureus* (MRSA) have been the main species of interest in veterinary research [1,2,3], antimicrobial-resistant non-*aureus* staphylococci (NAS), particularly methicillin-resistant NAS (MR-NAS), have also become a serious health issue in both humans and animals [4,5,6].

Frequent carriage of staphylococcal cassette chromosome *mec* type V (SCC*mec* V) has been observed in MRSA and MR-NAS isolates derived from livestock and farm environments, particularly in pigs and pig farm environments [7,8,9,10]. Moreover, the dissemination of SCC*mec* V elements between different species of staphylococci has been suggested [11,12]. Thus, NAS species are widely considered key reservoirs of various types of SCC*mec* elements [13,14]. In addition to SCC*mec* elements, NAS species can acquire diverse antimicrobial-resistance genes, leading to the emergence of multidrug-resistance (MDR) phenotypes against commonly used antimicrobial agents in livestock animals, such as β-lactams, phenicols, tetracyclines, and fluoroquinolones [15,16]. Notably, the number of reports of NAS isolates carrying the chloramphenicol–florfenicol resistance gene (*cfr*) have increased [17,18]. The *cfr* confers resistance to linezolid (LZD), which is a drug of last resort for treating infections caused by vancomycin-resistant enterococci and MRSA [19]. The first identification of *cfr* gene in a bovine-associated *Staphylococcus sciuri* isolate also suggests that NAS species acts as important reservoirs of critical AMR genes [20]. Therefore, putative transmission of AMR genes to and from staphylococci poses a significant threat to animal health by causing hard-to-treat infections that reduce productivity and welfare, while their zoonotic potential raises serious public health concerns.

*Staphylococcus borealis* is a recently recognized coagulase-negative NAS species that has frequently been misidentified as *Staphylococcus haemolyticus* owing to genotypic and phenotypic similarities [21,22]. Considering the initial reclassification of *S. borealis* isolates recovered from the human skin and blood in 2020 [21], its clinical significance has since been highlighted by reports of isolation from immunocompromised patients, suggesting its role as an opportunistic pathogen [22]. In addition, *S. borealis* has also been isolated from livestock, notably from bovine mastitis cases and from pigs, underscoring the importance of investigating livestock-derived isolates. In Spain, methicillin-resistant *S. borealis* (MRSB) carrying SCC*mec* V was isolated from healthy pigs [23] and was suggested as a potential zoonotic pathogen capable of spreading AMR genes among different NAS species. However, information regarding the AMR profiles and genetic factors associated with the AMR phenotypes of livestock-derived *S. borealis* isolates is limited.

Therefore, in this study, 92 *S. borealis* isolates were collected from 20 different pig farms (pigs, farm environments, and farmers) in South Korea to investigate: (i) their AMR profiles; (ii) the prevalence of MRSB and profiles of SCC*mec* types; (iii) the occurrence of *cfr*-mediated LZD resistance; and (iv) the genomic structure of a *cfr*-positive MRSB isolate via whole-genome sequence (WGS) analysis.

## 2. Results

### 2.1. Prevalence of S. borealis in Pig Farms in Korea

**Figure 1:**
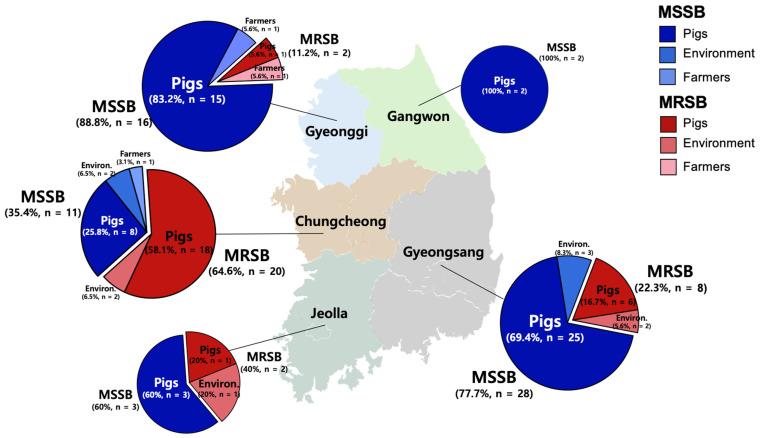
Distribution of MSSB and MRSB isolates from pigs, farm workers, and farm environments in Korea.

As presented in Table 1, 92 *S. borealis* isolates were obtained from pigs (n = 79), farm workers (n = 3), and farm environments (n = 10). Although the overall rate of methicillin-resistant *S. borealis* (MRSB) in 92 isolates was 34.8% (32/92 isolates), 81.3% (26/32 isolates) of the MRSB isolates were derived from healthy pigs (Figure 1). However, one MRSB isolate was identified in a pig farmer in Gyeonggi. The isolation rates of MRSB were 3.4%, 2.9%, and 2.3% in pigs, farm workers, and farm environments, respectively.

The overall prevalence of methicillin-susceptible *S. borealis* (MSSB) was higher than that of MRSB. The isolation rates of MSSB were 7.0%, 5.9%, and 2.3% in pigs, farm workers, and farm environments, respectively (Table 1).

### 2.2. AMR Profiles of S. borealis Isolates

As shown in Table 2, all 92 *S. borealis* isolates were susceptible to ciprofloxacin (CIP), mupirocin (MUP), rifampicin (RIF), and LZD except for one pig-associated CIP-resistant MSSB isolate. The MRSB isolates (96.9%) exhibited considerably higher levels of MDR phenotypes than those of the MSSB isolates (50.0%). Both MRSB and MSSB isolates showed >80% of resistance phenotypes to chloramphenicol (CHL) and clindamycin (CLI), both of which are classified as highly important antimicrobials for human and veterinary medicine. In addition, both MRSB and MSSB isolates showed similar resistance profiles regardless of the three different sample sites (pigs, farm workers, and farm environments).

### 2.3. High Prevalence of SCCmec V Among MRSB Isolates

**Figure 2:**
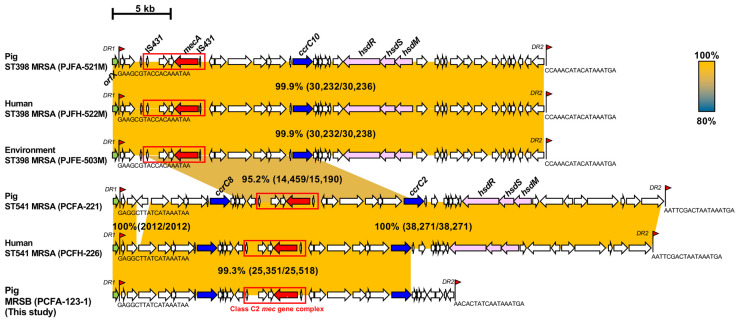
Comparative analysis of SCC*mec* V elements from MRSB isolated from pig farms. The SCC*mec* V sequences in the MRSB strain (PCFA-123-1) were compared with those of five MRSA strains available in the GenBank database: ST398 MRSA strain PJFA-521M (SRKD00000000), ST398 MRSA strain PJFH-522M (RKRI00000000), ST398 MRSA strain PJFE-503M (CP049976-CP049977), ST541 MRSA strain PCFA-221 (CP035003-CP035004), and ST541 MRSA strain PCFH-226 (CP035005-CP035006). SCC*mec* V, staphylococcal cassette chromosome methicillin resistance gene type V; MRSB, methicillin-resistant *S. borealis*; MRSA, methicillin-resistant *S. aureus*. Homologous regions are marked with colors corresponding to nucleotide sequence identities, and their percentages are indicated. The light green arrow indicates *orfX*. The methicillin resistance (*mec*) and chromosomal cassette recombinase (*ccr*) genes are shaded red and blue, respectively. The class C2 *mec* gene complex, composed of IS*431*-*mecA*-*ΔmecR1*::IS*431*, is specifically highlighted with a red box. The type I restriction-modification system composed of hydroxysteroid dehydrogenase genes (*hsdR*, *hsdS*, and *hsdM*) is shaded pink.

All 32 MRSB isolates were *mecA*-positive and exhibited cefoxitin (FOX) resistance (Table 3). SCC*mec* type analysis of the MRSB isolates revealed that all 32 isolates carried SCC*mec* V for the methicillin-resistance phenotype. WGS analysis of the MRSB PCFA-123-1 isolate revealed that PCFA-123-1 possessed 30 kb-sized SCC*mec* V integrated into the *orfX* regions. SCC*mec* V in the PCFA-123-1 isolate were consisted of a single compartment divided by two direct repeats (Figure 2). Comparison of the SCC*mec* V sequences of the PCFA-123-1 isolate with those of five MRSA strains (three ST398 MRSA and two ST541 MRSA isolates) revealed that all six SCC*mec* elements contained the class C2 *mec* gene complex and type 5 *ccrC* gene complex, thereby suggesting a prototype of SCC*mec* V (5C2 in ST398 and 5C2 and 5 in ST541). Similar to those of the two ST541 MRSA strains (PCFA-221 and PCFH-226), PCFA-123-1 harbored two type 5 *ccrC* genes (*ccrC2* and *ccrC8*), displaying the highest nucleotide sequence homology (99.3%) to that of the ST541 MRSA strain (PCFH-226). In contrast, three ST398 MRSA strains (PJFA-521M, PJFH-522M, and PJFE-503) strains carried a single type 5 *ccrC* gene (*ccrC10*) and class C2 *mec* gene complex, thus sharing 95.2% of nucleotide sequence identity with an ST541 MRSA (PCFA-221).

### 2.4. Identification of cfr-Positive Isolates and Comparative Analysis of cfr-Containing Regions

As shown in Table 3, 62.5% (20/32) of MRSB isolates and 3.3% (2/60) of MSSB isolates were *cfr* positive. None of the 22 *cfr*-positive *S. borealis* isolates showed resistance phenotype to LZD (Table 2 and Table 3).

**Figure 3:**
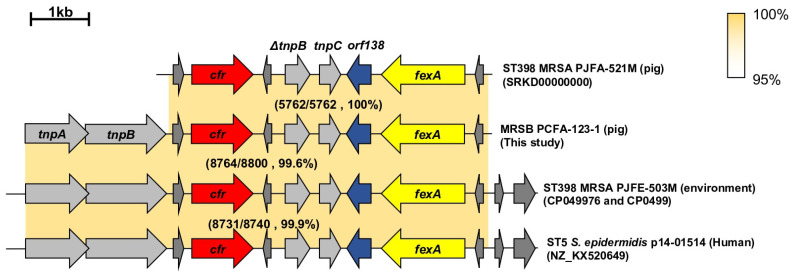
Comparative analysis of the *cfr*-containing regions from MRSB isolated from a pig farm. Arrows indicate the positions and orientations of the genes. Nucleotide sequences >95% of similarity are shown in yellow.

WGS analysis of the *cfr*-positive but LZD-susceptible MRSB isolate (PCFA-123-1) revealed that *cfr* was carried on a 38 kb-plasmid. Comparative analysis of the *cfr*-containing genetic regions in the MRSB and the previously sequenced plasmids of ST398 MRSA strains [24,25] and *S. epidermidis* [26] revealed that all strains carried the florfenicol–chloramphenicol exporter gene (*fexA*) downstream of the *cfr* (Figure 3). Except for the PJFA-521M strain, all three *cfr*-positive strains carried two transposase genes (*tnpA* and *tnpB*), which are associated with the mobility of Tn*558*. The *cfr*-containing genetic regions of the *S. borealis* PCFA-123-1 isolate shared 100% sequence similarity with that of the ST398 MRSA strain (PJFA-521M).

## 3. Discussion

Although *S. borealis* is a relatively newly recognized coagulase-negative NAS, concerns regarding AMR of *S. borealis* isolates are increasing. Antimicrobial-resistant *S. borealis* has been identified in various hosts, including human patients, bovine mammary glands, and pigs [22,23,27]. However, data regarding the prevalence and AMR profiles of *S. borealis* isolates associated with livestock farms is limited.

This study describes the prevalence and AMR profiles of *S. borealis* isolates collected from pigs, farm workers, and farm environments in Korean pig farms. The overall isolation rate of *S. borealis* was 9.1% (92 isolates from 1009 swabs), and 34.8% (32/92) of *S. borealis* isolates were MRSB (Table 1). The rate of methicillin resistance (34.8%) in *S. borealis* isolates observed in this study was comparable with that of *S. aureus* isolates (40/81, 49.4%) and *S. epidermidis* isolates (22/89, 24.7%) obtained from pig farms in Korea in previous studies [28,29]. Although 79/92 *S. borealis* isolates were obtained from pigs in this study, three isolates (one MRSB and two MSSB) were obtained from nasal swabs of farm workers. Although only three isolates from farm workers were included in this study, this result indicates potential zoonotic transmission of pig-associated *S. borealis* to human workers, most likely facilitated by frequent contact with pigs or indirect exposure to contaminated aerosols or feeding areas in the pig farms.

Both the MRSB and MSSB isolates exhibited the highest resistance to chloramphenicol (CHL) and clindamycin (CLI), which correlated with the frequent and prolonged use of these antimicrobial agents in pig farms in Korea [30,31]. This result also indicates the risk of reduced treatment efficacy and zoonotic transmission of resistant strains. Moreover, the MRSB isolates showed higher levels of resistance to ampicillin (AMP), sulfamethoxazole-trimethoprim (SXT), and tetracycline (TET) than those of MSSB isolates. This contributed to the increased prevalence of the MDR phenotype in MRSB isolates (Table 2). Resistance to these antimicrobials is commonly associated with genes such as *cfr*/*fexA* (CHL), *erm* (CLI), *blaZ*/*mecA* (AMP), *dfr*/*sul* (SXT), and *tet* genes (TET), which are frequently carried on mobile genetic elements that facilitate horizontal transfer. The AMR profiles of the MRSB isolates observed in the current study were similar to those of MRSA [28] and methicillin-resistant *S. epidermidis* (MRSE) isolates [29] obtained from Korean pig farms in previous studies. These results suggest that *S. borealis* strains originating from pigs can also serve as reservoirs of AMR and consequently present as a critical public health concern.

Previous studies reported a high prevalence of SCC*mec* V among MRSA and methicillin-resistant NAS isolates derived from Korean livestock farms, particularly pig farms [11,28,29,32]. SCC*mec* V has been frequently identified in the clonal complex (CC) 398 lineages of LA-MRSA isolates, which is the most commonly reported LA-MRSA clone in many countries, including Korea [33,34]. Interestingly, all 32 MRSB isolates in this study carried SCC*mec* V for methicillin resistance (Table 3). In Spain, MDR *S. borealis* isolates carrying SCC*mec* V were also detected in healthy pigs. The co-occurrence of identical SCC*mec* V elements among the MRSA and MR-NAS isolates suggests the horizontal transmission of SCC*mec* V between different species of staphylococci in livestock farms, given that SCC*mec* is a mobile genetic element capable of interspecies transfer. As shown in Figure 2, WGS analysis of the MRSB isolate (PCFA-123-1) revealed two essential components of SCC*mec* V, a type 5 *ccrC* gene complex and a class C2 *mec* gene complex. Although the three ST398 MRSA strains carried one *ccrC* and a class C2 *mec* gene complex in SCC*mec* V, an additional *ccrC* gene was identified in the two ST541 MRSA and PCFA-123-1 isolates (Figure 2). Although structural variations were observed in the joining (J) regions of SCC*mec* V, the core regions comprising the class C2 *mec* gene complex and type 5 *ccrC* gene complexes in the MRSB strain showed >95% nucleotide sequence homology with the five MRSA strains isolated from pig farms. This provides molecular evidence that the highly conserved structure of SCC*mec* V has been transferred among *S. aureus* and *S. borealis* isolates from pig farms, which underscores the potential zoonotic risk and public health concerns.

As shown in Table 3, 22 of the 92 *S. borealis* isolates were *cfr*-positive. The *cfr* gene produces a ribosomal methyltransferase that leads to phenicols, lincosamides, oxazolidinones, pleuromutilins, and streptogramin A (PhLOPS_A_) resistance phenotype [35,36]. In addition to the SCC*mec* V, WGS analysis of the PCFA-123-1 isolate revealed that *cfr* was carried on a 38 kb-plasmid along with *fexA* gene (Figure 3). However, all 22 *cfr*-positive *S. borealis* isolates, including the PCFA-123-1 isolate, were susceptible to LZD (Table 2 and Table 3). A point mutation within the *cfr* open reading frame (ORF), Q148K, was identified in *cfr*-positive but LZD-susceptible *S. aureus* and NAS isolates from pig farms [25]. Thus, sequencing analyses of the *cfr* ORFs in the 22 *cfr*-positive *S. borealis* isolates were performed. However, no point mutations were identified in these isolates (Table S1). Furthermore, a 35-bp insertion sequence in the *cfr* promoter region was found to be responsible for the LZD-susceptible phenotype in *cfr*-positive *S. aureus* isolates [37]. Sequencing analyses of the *cfr* promoter region revealed that 19 of the 22 *cfr*-positive *S. borealis* isolates, including the PCFA-123-1 isolate, harbored the previously described 35-bp insertion sequences [37]. This indicated that the plasmids carrying the 35-bp insertion mutations were transmitted between *S. aureus* and NAS isolates in the pig farms. Interestingly, the remaining three isolates displayed a wild-type *cfr* ORFs and promoter sequences, but were still susceptible to LZD. In a previous study, it has also been reported that linezolid-susceptible phenotype may emerge even in the presence of the wild-type *cfr* gene in CoNS [23]. Comparative analysis of the plasmid regions containing *cfr* genes also confirmed carriage of *cfr* and *fexA* in the plasmids of *S. borealis*, *S. aureus*, and *S. epidermidis,* with >99.6% nucleotide sequence homology with the p14-01514 plasmid in a clinical strain of *S. epidermidis* (Figure 3). Taken together, the presence of antimicrobial resistance genes within mobile genetic elements highlights the role of *S. borealis* in horizontal transmission of AMR in livestock farms.

## 4. Materials and Methods

### 4.1. Swab Samples and Isolation of S. borealis

A total of 1009 swab samples were collected from 20 different pig farms located in five provinces of South Korea in 2017. Swab samples were collected from pigs (760 nasal swabs), farm environments (215 swabs from floors, sewage areas, ventilators, and fences), and farm workers (34 nasal swabs). Sampling was performed using sterile cotton swabs (Copan Italia Spa, Brescia, Italy). All swab samples were placed into sterile transport tubes, stored at 4 °C in ice-cooled containers, and transported to the laboratory for bacterial isolation within 24 h. The sampling procedure was reviewed and approved by the Institutional Review Board (NHIMC 2017-07-041) and Institutional Animal Care and Use Committee (2017-00112).

For NAS isolation, swab samples from pig farms were inoculated into 3.5 mL of tryptic soy broth (Difco Laboratories, Detroit, MI, USA) supplemented with 10% NaCl and incubated at 37 °C for 18–24 h. Thereafter, ~20-µL aliquots of the pre-enriched cultures were streaked onto Baird-Parker agar (Difco Laboratories) containing 5% egg yolk and potassium tellurite. The plates were then incubated at 37 °C for up to 48 h. Presumptive staphylococcal colonies were selected and re-streaked on tryptic soy agar for subsequent experiment. *S. borealis* was identified using matrix-assisted laser desorption ionization time-of-flight mass spectrometry (MALDI-TOF/MS; Bruker Daltonics GmbH, Bremen, Germany) with the MBT reference library v12.0 and 16S ribosomal RNA sequencing. Sequence analysis of *hsp60* was performed to distinguish *S. borealis* from *S. haemolyticus* as previously described [21].

### 4.2. Antimicrobial Susceptibility Test

The AMR profiles of *S. borealis* isolates were analyzed using the standard disc diffusion method according to the Clinical and Laboratory Standards Institute’s (CLSI) guidelines [38]. Briefly, each isolate was suspended in 0.85% saline and evenly swabbed onto Mueller–Hinton agar (Difco Laboratories) plates. Antimicrobial discs were then placed on the agar surface, and plates were incubated at 37 °C. Following incubation, the diameters of inhibition zones were measured, and susceptibility or resistance was interpreted according to the CLSI breakpoints. The following 13 antimicrobial agents were used: ampicillin (AMP, 10 μg), cefoxitin (FOX, 30 μg), ciprofloxacin (CIP, 5 μg), clindamycin (CLI, 2 μg), chloramphenicol (CHL, 30 μg), erythromycin (ERY, 15 μg), gentamicin (GEN, 30 μg), linezolid (LZD), mupirocin (MUP, 200 μg), quinupristin–dalfopristin (SYN, 15 μg), rifampin (RIF, 5 μg), sulfamethoxazole-trimethoprim (SXT, 23.73–1.25 μg), and tetracycline (TET, 30 μg). All antimicrobial agents used for the disc diffusion assay, except MUP (Oxoid, Hampshire, UK), were obtained from BD BBLTM (Becton Dickinson, Franklin Lakes, NJ, USA). The *S. aureus* ATCC 29213 strain was used as a reference strain for the disc diffusion tests.

### 4.3. SCCmec Typing and Detection of cfr

*S. borealis* isolates showing FOX resistance phenotype were screened for the presence of *mecA* gene using polymerase chain reaction (PCR), as described previously [39]. For all *mecA*-positive *S. borealis* isolates, SCC*mec* types were determined by PCR amplification of *mec* regulatory elements (*mec*) and chromosomal cassette recombinase (*ccr*), and subsequent assignment of SCC*mec* types was performed as previously described [40].

The presence of *cfr*, which confers LZD resistance by encoding a 23S rRNA methyltransferase [41], was screened for all *S. borealis* isolates using the PCR method as described before [36].

### 4.4. WGS Analysis

A *cfr*-positive *S. borealis* PCFA-123-1 isolate carrying SCC*mec* V for methicillin resistance was subjected to WGS analysis using a TruSeq DNA PCR-free kit (Illumina Inc., San Diego, CA, USA). The concentration and purity of the DNA sample was assessed using a NanoDrop^TM^ 2000c spectrophotometer (Thermo Fisher Scientific, Wilmington, NC, USA) as previously described [42], and 1.0 μg of total DNA was used for the library construction. Genomic sequences of the *S. borealis* isolates were generated using a combination of the Illumina iSeq platform (Illumina) and Oxford Nanopore MinION (Oxford Nanopore Technologies, Oxford, UK). The raw reads generated via 150-bp paired-end sequencing on the Illumina platform were trimmed using Trimmomatic (v0.36) to eliminate low-quality adaptor sequences. Raw read sequencing data were *de novo* assembled using SPAdes (v3.13), and library preparation was carried out using MinION reads in Trycycler v.0.3.0 (https://github.com/rrwick/Trycycler (accessed on 2 June 2020)). Rapid Annotation was carried out using Subsystem Technology and functional annotation was performed using Prokka (v1.12).

SCC*mec*Finder v1.2 (https://cge.food.dtu.dk/services/SCCmecFinder/ (accessed on 13 July 2020)) of the Center for Genomic Epidemiology (CGE) (http://www.genomicepidemiology.org/ (accessed on 13 July 2020)) was used to analyze SCC*mec* elements by identifying the combination of *mec* and *ccr* gene complexes within assembled contigs and comparing them with reference SCC*mec* structures. The locations of the *cfr* gene and other AMR genes were analyzed using the Comprehensive Antibiotic Resistance Database (https://card.mcmaster.ca/ (accessed on 13 July 2020)) and ResFinder v4.1 of the CGE databases with resistance determinants assigned based on sequence identity (≥90%) relative to reference sequences.

### 4.5. Comparative Analysis of SCCmec V and cfr

For comparative analysis of the SCC*mec* V elements in the PCFA-123-1 isolate, raw sequence files of three ST398 MRSA isolates (ST398 PJFA-521M from a pig [GenBank accession no. SRKD00000000] [24], ST398 PJFH-522M from a pig farm worker [GenBank accession no. RKRI00000000], ST398 PJFE-503M from pig farm environment [GenBank accession no. CP049976-CP049977] [25]) and two ST541 isolates (ST541 PCFA-221 from a pig [GenBank accession no. CP035003-CP035004] and ST541 PCFH-226 from a pig farm worker [GenBank accession no. CP035005-CP035006]) carrying SCC*mec* V were included in the analyses. The conserved *orfX* site where insertion and excision of SCC*mec* occur, along with its flanking direct repeat regions, was used to identify SCC*mec* structures in each genome. The nucleotide sequences and gene organizations of the SCC*mec* elements were then compared using BLAST version 5 to assess structural similarity and homology across isolates.

To examine the *cfr*-containing genomic region in the *S. borealis* isolate, comparative sequence analysis of PCFA-123-1 and previously reported *cfr*-positive staphylococci was performed using BLAST to assess sequence conservation and gene organization. These strains included two ST398 LA-MRSA strains (PJFA-521M and PJFE-503M) [24,25] and the ST5 *S. epidermidis* p14-01514 (GenBank accession no. NZ_KX520649) [26].

### 4.6. Nucleotide Sequence Accession Number

The WGS data of *S. borealis* PCFA-123-1 were deposited in the NCBI database under the accession numbers of CP116211-CP116213.

### 4.7. Statistical Analysis

All quantitative data were analyzed using the Kruskal–Wallis test for multiple-group comparisons with Dunn’s post hoc test (IBM SPSS Statistics 25, Chicago, IL, USA). A *p*-value < 0.05 was considered statistically significant.

## 5. Conclusions

In conclusion, our results suggest that (i) *S. borealis* isolates with MDR phenotypes colonize healthy pigs, farm workers, and farm environments; (ii) SCC*mec* V is predominantly distributed in pig-associated MRSB isolates in Korea; (iii) the 35-bp insertion sequence in the *cfr* promoter region is responsible for LZD-susceptibility in most *cfr*-positive *S. borealis* isolates; and (iv) different species of staphylococci, including *S. aureus* and *S. borealis*, acquire SCC*mec* V- and *cfr*-containing plasmids through intra- and inter-species transmission in pig farms. It should be recognized that the present study has some limitations. First, only three isolates obtained from farm workers limits the representativeness of human-associated *S. borealis*. Second, the comparative analyses of SCC*mec* V- and *cfr*-containing plasmids were performed using a single representative *S. borealis* isolate (PCFA-123-1). However, our findings provide important insights into the AMR profiles and genetic determinants of livestock-associated *S. borealis*, underscoring their implications for both animal and human health. Therefore, continuous monitoring of AMR in livestock-associated staphylococci is essential to limit further spread and protect public health.

## Figures and Tables

**Table 1:** *S. borealis* isolates collected from pigs, farm workers, and farm environments.

	No. of Isolates/No. of Samples (%)		
*S. borealis* (n = 92)	Pig Farms (n = 92/1009, 9.1%)		
	Pigs	Farmers	Environ. ^1^
MRSB ^2^ (n = 32/92)	26/760 (3.4%)	1/34 (2.9%)	5/215 (2.3%)
MSSB ^3^ (n = 60/92)	53/760 (7.0%)	2/34 (5.9%)	5/215 (2.3%)
Total	79/760 (10.4%)	3/34 (8.8%)	10/215 (4.6%)

^1^ Environ., environments. ^2^ MRSB, methicillin-resistant *S. borealis*. ^3^ MSSB, methicillin-susceptible *S. borealis*.

**Table 2:** Antimicrobial resistance profiles of 92 *S. borealis* isolates recovered from pigs, farm workers, and farm environments.

		Number (%) of Isolates Resistant to ^1^													
		AMP	FOX	CHL	CIP	CLI	ERY	GEN	MUP	RIF	SXT	SYN	TET	LZD	MDR
MRSB ^2^ (n = 32)	Pigs (n = 26)	26 (100)	26 (100)	25 (96.2)	0	26 (100)	0	5 (19.2)	0	0	20 (76.9)	2 (7.7)	25 (96.2)	0	26 (100)
	Farmers (n = 1)	1 (100)	1 (100)	1 (100)	0	1 (100)	1 (100)	1 (100)	0	0	0	0	1 (100)	0	1 (100)
	Environ. ^4^ (n = 5)	4 (80)	5 (100)	4 (80)	0	4 (80)	0	0	0	0	2 (40)	0	4 (80)	0	4 (80)
	Total	31 (96.9)	32 (100)	30 (93.8)	0	31 (96.9)	1 (3.1)	6 (18.8)	0	0	22 (68.8)	2 (6.3)	30 (93.8)	0	31 (96.9)
MSSB ^3^ (n = 60)	Pigs (n = 53)	2 (3.8)	0	41 (77.4)	1 (1.9)	44 (83)	26 (49.1)	1 (1.9)	0	0	0	1 (1.9)	21 (39.6)	0	27 (50.9)
	Farmers (n = 2)	0	0	2 (100)	0	2 (100)	0	0	0	0	0	0	0	0	0
	Environ. (n = 5)	0	0	5 (100)	0	5 (100)	3 (60)	0	0	0	0	0	3 (60)	0	3 (60)
	Total	2 (3.3)	0	48 (80)	1 (1.7)	51 (85)	29 (48.3)	1 (1.7)	0	0	0	1 (1.7)	24 (40)	0	30 (50)
Total (n = 92)		33 (35.9)	32 (34.8)	78 (84.8)	1 (1.1)	82 (89.1)	30 (32.6)	7 (7.6)	0	0	22 (23.9)	3 (3.3)	54 (58.7)	0	61 (66.3)

^1^ AMP, ampicillin; FOX, cefoxitin; CHL, chloramphenicol; CIP, ciprofloxacin; CLI, clindamycin; ERY, erythromycin; GEN, gentamicin; MUP, mupirocin; RIF, rifampicin; SXT, trimethoprim-sulfamethoxazole; SYN, quinupristin–dalfopristin; TET, tetracycline; LZD, linezolid; MDR, multidrug resistance. ^2^ MRSB, methicillin-resistant *S. borealis*. ^3^ MSSB, methicillin-susceptible *S. borealis*. ^4^ Environ., environment.

**Table 3:** Carriage of antimicrobial resistance genes in MRSB and MSSB isolates from pig farms in Korea.

		No. of *S. borealis* Isolates		
		Methicillin Resistance		Carriage of *cfr*
MR/MS		*mecA*	SCC*mec* Type	
MRSB ^1^ (n = 32)	Pigs (n = 26)	26/26 (100%)	SCC*mec* V (26/26, 100%) SCC*mec* V (1/1, 100%) SCC*mec* V (5/5, 100%)	17/26 (65.4%)
	Farmers (n = 1)	1/1 (100%)		1/1 (100%)
	Environ. ^3^ (n = 5)	5/5 (100%)		2/5 (40.0%)
	Total	32/32 (100%)	32/32 (100%)	20/32 (62.5%)
MSSB ^2^ (n = 60)	Pigs (n = 53)	-	-	2/53 (3.8%)
	Farmers (n = 2)	-	-	-
	Environ. (n = 5)	-	-	-
	Total	-	-	2/60 (3.3%)
Total (n = 92)		32/92 (34.8%)		22/92 (23.9%)

^1^ MRSB, methicillin-resistant *S. borealis*; ^2^ MSSB, methicillin-susceptible *S. borealis*; ^3^ Environ., environment.

## Data Availability

No new data were created or analyzed in this study. Data sharing is not applicable to this article.

## Supplementary Materials

The following supporting information can be downloaded at https://www.mdpi.com/article/10.3390/antibiotics14090910/s1, Table S1: Genetic mutation of *cfr* and linezolid resistance phenotypes of *cfr*-carrying *S. borealis*.

## References

[1] Fluit A. (2012). Livestock-associated *Staphylococcus aureus*. Clin. Microbiol. Infect..

[2] Fitzgerald J.R. (2012). Livestock-associated *Staphylococcus aureus*: Origin, evolution and public health threat. Trends Microbiol..

[3] da Silva A.C., Rodrigues M.X., Silva N.C.C. (2020). Methicillin-resistant *Staphylococcus aureus* in food and the prevalence in Brazil: A review. Braz. J. Microbiol..

[4] Argudín M.A., Vanderhaeghen W., Butaye P. (2015). Antimicrobial resistance and population structure of *Staphylococcus epidermidis* recovered from pig farms in Belgium. Vet. J..

[5] Vanderhaeghen W., Vandendriessche S., Crombé F., Dispas M., Denis O., Hermans K., Haesebrouck F., Butaye P. (2012). Species and staphylococcal cassette chromosome *mec* (SCC*mec*) diversity among methicillin-resistant non-*Staphylococcus aureus* staphylococci isolated from pigs. Vet. Microbiol..

[6] Osman K., Badr J., Al-Maary K.S., Mousse I.M.I., Hessain A.M., Girah Z.M.S.A., Abo-shamas U.H., Orabi A., Saad A. (2016). Prevalence of the antibiotic resistance genes in coagulase-positive-and negative-*Staphylococcus* in chicken meat retailed to consumers. Front. Microbiol..

[7] Price L.B., Stegger M., Hasman H., Aziz M., Larsen J., Andersen P.S., Pearson T., Waters A.E., Foster J.T., Schupp J. (2012). *Staphylococcus aureus* CC398: Host adaptation and emergence of methicillin resistance in livestock. mBio.

[8] Pirolo M., Gioffrè A., Visaggio D., Gherardi M., Pavia G., Samele P., Ciambrone L., Di Natale R., Spatari G., Casalinuovo F. (2019). Prevalence, molecular epidemiology, and antimicrobial resistance of methicillin-resistant *Staphylococcus aureus* from swine in southern Italy. BMC Microbiol..

[9] Venugopal N., Mitra S., Tewari R., Ganaie F., Shome R., Rahman H., Shome B.R. (2019). Molecular detection and typing of methicillin-resistant *Staphylococcus aureus* and methicillin-resistant coagulase-negative staphylococci isolated from cattle, animal handlers, and their environment from Karnataka, Southern Province of India. Vet. World.

[10] Feßler A.T., Kadlec K., Hassel M., Hauschild T., Eidam C., Ehricht R., Monecke S., Schwarz S. (2011). Characterization of methicillin-resistant *Staphylococcus aureus* isolates from food and food products of poultry origin in Germany. Appl. Environ. Microbiol..

[11] Tulinski P., Fluit A.C., Wagenaar J.A., Mevius D., van de Vijver L., Duim B. (2012). Methicillin-resistant coagulase-negative staphylococci on pig farms as a reservoir of heterogeneous staphylococcal cassette chromosome *mec* elements. Appl. Environ. Microbiol..

[12] Takahashi T., Kim H., Kim H.S., Kim H.S., Song W., Kim J.S. (2024). Comparative genomic analysis of staphylococcal cassette chromosome *mec* type V *Staphylococcus aureus* strains and estimation of the emergence of SCC*mec* V clinical isolates in Korea. Ann. Lab. Med..

[13] Wolska-Gębarzewska M., Międzobrodzki J., Kosecka-Strojek M. (2024). Current types of staphylococcal cassette chromosome *mec* (SCC *mec*) in clinically relevant coagulase-negative staphylococcal (CoNS) species. Crit. Rev. Microbiol..

[14] Saber H., Jasni A.S., Jamaluddin T.Z.M.T., Ibrahim R. (2017). A review of staphylococcal cassette chromosome *mec* (SCC*mec*) types in coagulase-negative staphylococci (CoNS) species. Malays. J. Med. Sci..

[15] Srednik M.E., Tremblay Y.D., Labrie J., Archambault M., Jacques M., Fernández Cirelli A., Gentilini E.R. (2017). Biofilm formation and antimicrobial resistance genes of coagulase-negative staphylococci isolated from cows with mastitis in Argentina. FEMS Microbiol. Lett..

[16] Rossi C.C., Pereira M.F., Giambiagi-deMarval M. (2020). Underrated *Staphylococcus* species and their role in antimicrobial resistance spreading. Genet. Mol. Biol..

[17] Cuny C., Arnold P., Hermes J., Eckmanns T., Mehraj J., Schoenfelder S., Ziebuhr W., Zhao Q., Wang Y., Feßler A.T. (2017). Occurrence of *cfr*-mediated multiresistance in staphylococci from veal calves and pigs, from humans at the corresponding farms, and from veterinarians and their family members. Vet. Microbiol..

[18] Ruiz-Ripa L., Feßler A.T., Hanke D., Sanz S., Olarte C., Mama O.M., Eichhorn I., Schwarz S., Torres C. (2020). Coagulase-negative staphylococci carrying *cfr* and PVL genes, and MRSA/MSSA-CC398 in the swine farm environment. Vet. Microbiol..

[19] Chen H., Du Y., Xia Q., Li Y., Song S., Huang X. (2020). Role of linezolid combination therapy for serious infections: Review of the current evidence. Eur. J. Clin. Microbiol. Infect. Dis..

[20] Schwarz S., Werckenthin C., Kehrenberg C. (2000). Identification of a plasmid-borne chloramphenicol-florfenicol resistance gene in *Staphylococcus sciuri*. Antimicrob. Agents Chemother..

[21] Pain M., Wolden R., Jaén-Luchoro D., Salvà-Serra F., Iglesias B.P., Karlsson R., Klingenberg C., Cavanagh J.P. (2020). *Staphylococcus borealis* sp. nov., isolated from human skin and blood. Int. J. Syst. Evol. Microbiol..

[22] Cavanagh J.P., Klingenberg C., Venter H.J., Afset J.E., Stromme O., Lindemann P.C., Johansen T., Zaragkoulias K., Aamot H.V., Tofteland S. (2025). Revealing the clinical relevance of *Staphylococcus borealis*. Microbiol. Spectr..

[23] Abdullahi I.N., Lozano C., Simón C., Zarazaga M., Torres C. (2023). Within-host diversity of coagulase-negative staphylococci resistome from healthy pigs and pig farmers, with the detection of *cfr*-carrying strains and MDR-*S. borealis*. Antibiotics.

[24] Lee G.Y., Seong H.J., Sul W.J., Yang S.J. (2021). Genomic information on linezolid-resistant sequence-type 398 livestock-associated methicillin-resistant *Staphylococcus aureus* isolated from a pig. Foodborne Pathog. Dis..

[25] Lee G.Y., Kim G.B., Yang S.J. (2022). Co-occurrence of *cfr*-mediated linezolid-resistance in ST398 LA-MRSA and non-*aureus* staphylococci isolated from a pig farm. Vet. Microbiol..

[26] Weßels C., Strommenger B., Klare I., Bender J., Messler S., Mattner F., Krakau M., Werner G., Layer F. (2018). Emergence and control of linezolid-resistant *Staphylococcus epidermidis* in an ICU of a German hospital. J. Antimicrob. Chemother..

[27] Król J., Wanecka A., Twardoń J., Florek M., Marynowska M., Banaszkiewicz S., Kaczmarek-Pieńczewska A., Pląskowska E., Brodala M., Chwirot W. (2023). *Staphylococcus borealis*–A newly identified pathogen of bovine mammary glands. Vet. Microbiol..

[28] Back S.H., Eom H.S., Lee H.H., Lee G.Y., Park K.T., Yang S.J. (2020). Livestock-associated methicillin-resistant *Staphylococcus aureus* in Korea: Antimicrobial resistance and molecular characteristics of LA-MRSA strains isolated from pigs, pig farmers, and farm environment. J. Vet. Sci..

[29] Lee G.Y., Lee H.H., Yang S.J. (2023). Antimicrobial resistance profiles and clonal diversity of *Staphylococcus epidermidis* isolates from pig farms, slaughterhouses, and retail pork. Vet. Microbiol..

[30] Scott H.M., Acuff G., Bergeron G., Bourassa M.W., Gill J., Graham D.W., Kahn L.H., Morley P.S., Salois M.J., Simjee S. (2019). Critically important antibiotics: Criteria and approaches for measuring and reducing their use in food animal agriculture. Ann. N. Y. Acad. Sci..

[31] Lim S.K., Lee J.E., Lee H.S., Nam H.M., Moon D.C., Jang G.C., Park Y.J., Jung Y.G., Jung S.C., Wee S.H. (2014). Trends in antimicrobial sales for livestock and fisheries in Korea during 2003–2012. Korean J. Vet. Res..

[32] Moon D.C., Jeong S.K., Hyun B.H., Lim S.K. (2019). Prevalence and characteristics of methicillin-resistant *Staphylococcus aureus* isolates in pigs and pig farmers in Korea. Foodborne Pathog. Dis..

[33] Wang Y., Zhang P., Wu J., Chen S., Jin Y., Long J., Duan G., Yang H. (2023). Transmission of livestock-associated methicillin-resistant *Staphylococcus aureus* between animals, environment, and humans in the farm. Environ. Sci. Pollut. Res..

[34] Lee S.I., Lee G.Y., Park J.H., Yang S.J. (2023). High prevalence of clonal complex 398 methicillin-susceptible and-resistant *Staphylococcus aureus* in pig farms: Clonal lineages, multiple drug resistance, and occurrence of the staphylococcal cassette chromosome *mec* IX. Foodborne Pathog. Dis..

[35] Gao Y., Wang Z., Fu J., Cai J., Ma T., Xie N., Fan R., Zhai W., Feßler A.T., Sun C. (2022). Spreading of *cfr*-carrying plasmids among staphylococci from humans and animals. Microbiol. Spectr..

[36] Lee G.Y., Yang S.J. (2023). Occurrence of *cfr*-positive linezolid-susceptible *Staphylococcus aureus* and non-*aureus* Staphylococcal isolates from pig farms. Antibiotics.

[37] Lee J.B., Lim J.H., Park J.H., Lee G.Y., Park K.T., Yang S.J. (2024). Genetic characteristics and antimicrobial resistance of *Staphylococcus aureus* isolates from pig farms in Korea: Emergence of *cfr*-positive CC398 lineage. Bmc Vet. Res..

[38] Clinical and Laboratory Standard Institute (CLSI) (2021). VET01S Performance Standards for Antimicrobial Disk and Dilution Susceptibility Tests for Bacteria Isolated from Animals.

[39] Geha D.J., Uhl J.R., Gustaferro C.A., Persing D.H. (1994). Multiplex PCR for identification of methicillin-resistant staphylococci in the clinical laboratory. J. Clin. Microbiol..

[40] Kondo Y., Ito T., Ma X.X., Watanabe S., Kreiswirth B.N., Etienne J., Hiramatsu K. (2007). Combination of multiplex PCRs for staphylococcal cassette chromosome *mec* type assignment: Rapid identification system for *mec*, *ccr*, and major differences in junkyard regions. Antimicrob. Agents Chemother..

[41] Tsai K., Stojković V., Noda-Garcia L., Young I.D., Myasnikov A.G., Kleinman J., Palla A., Floor S.N., Frost A., Fraser J.S. (2022). Directed evolution of the rRNA methylating enzyme Cfr reveals molecular basis of antibiotic resistance. eLife.

[42] Naorem R.S., Urban P., Goswami G., Fekete C. (2020). Characterization of methicillin-resistant *Staphylococcus aureus* through genomics approach. 3 Biotech.

