# Supplementary material for: Antimicrobial Resistance of Staphylococcus borealis Isolated from Pig Farms: High Prevalence of SCCmec Type V and Emergence of cfr-Positive Isolates
Source: Antibiotics (Basel). 2025 Sep 9;14(9):910. doi: 10.3390/antibiotics14090910 (PMC12466872; doi:10.3390/antibiotics14090910)
Supplement: Supplementary file 1 [file antibiotics-14-00910-s001.zip › antibiotics-3844224-supplementary.pdf]

Table S1. Genetic mutation of *cfr* and linezolid resistance phenotypes of *cfr*-carrying *S. borealis*.

MR/MS	Source	Strains	Genetic mutation of *cfr*	Linezolid resistance (≥8)	
MRSB[a] (n = 20)	Pigs (n = 17)	PCFA-111	35bp insertion into the promoter	≤1	S
		PCFA-121		≤1	S
		PCFA-123-1		≤1	S
		PCFA-123-2		≤1	S
		PCFA-141		≤1	S
		PCFA-142		2	S
		PCFA-151		≤1	S
		PCFA-153		≤1	S
		PCFA-163		≤1	S
		PCFA-171		≤1	S
		PCFA-182		≤1	S
		PCFA-183		≤1	S
		PCFA-184		≤1	S
		PCFA-191		≤1	S
		PCFA-193		≤1	S
		PCFA-1102		≤1	S
		PCFA-1103		≤1	S
	Environ.[c] (n = 2)	PCFE-102		≤1	S
		PCFE-103		≤1	S
	Farmers (n = 1)	PSFH-341	Wild type	≤1	S
MSSB[b] (n = 2)	Pigs (n = 2)	PSFA-143	Wild type	≤1	S
		PSFA-152		≤1	S

[a]MRSB, methicillin-resistant *S. borealis*; [b]MSSB, methicillin-susceptible *S. borealis*; [c]Environ.; environment
